# A Quality Improvement Intervention to Enhance Documentation on Histopathology Request Forms

**DOI:** 10.7759/cureus.81317

**Published:** 2025-03-27

**Authors:** Farrukh Ansar, Mohammad S Rauf, Muhammad Kinwan Khan, Uzma Rauf, Muhammad Bilal Ahmad, Ayesha Ishtiaq, Muhammad Zuama Zafar Butt, Fatima Abdul Hameed, Sabahat Ali, Amna Amin

**Affiliations:** 1 Medicine, Alkhidmat Raazi Hospital, Rawalpindi, PAK; 2 Medicine, Northwest School of Medicine, Peshawar, PAK; 3 Surgery, Northwest General Hospital, Peshawar, PAK; 4 Medicine, St. Vincent Medical Center, Toledo, USA; 5 Medicine, Quaid-e-Azam International Hospital, Islamabad, PAK; 6 Surgery, Quaid-e-Azam International Hospital, Islamabad, PAK; 7 Emergency Medicine, Quaid-e-Azam International Hospital, Islamabad, PAK

**Keywords:** clinical audit, documentation errors, histopathology, pdsa cycle, quality improvement

## Abstract

Introduction

Incomplete histopathology request form documentation can compromise diagnostic accuracy and delay patient management. This study aimed to assess and improve documentation completeness using a structured quality improvement approach.

Methods

A clinical audit was conducted at a tertiary care hospital using the Plan-Do-Study-Act (PDSA) cycle. In the first audit cycle, 250 histopathology request forms were reviewed for completeness. Based on the findings, targeted interventions were implemented, including a standardized request form, clinician engagement, and improved accessibility to forms. A second audit cycle assessed 150 forms to evaluate the impact of these interventions.

Results

Significant improvements were observed across all documentation parameters. Clinical history documentation increased from 0% to 62%, while presenting complaints improved from 3.2% to 73%. Physical examination findings were recorded in 96% of cases compared to 73.6% initially, and radiological findings improved from 44.4% to 95%. Laboratory investigation results increased from 41.2% to 81%, while drug/medication history documentation rose from 6% to 48%. Specimen details also showed improvement, with biopsy time documentation increasing from 3.2% to 66% and provisional diagnosis documentation rising from 49.2% to 78%.

Conclusion

A structured quality improvement approach led to significant enhancements in documentation completeness. Ongoing audits, clinician training, and digital solutions are recommended for sustaining these improvements.

## Introduction

Modern medical practice is becoming increasingly reliant on reliable clinical laboratory, radiological, and histopathological services that can determine the fate of a treatment plan's course of action [1]. Histopathology is an essential part of medical diagnostics, providing critical information about diseases to help in diagnosis, treatment, and patient management [2]. The histopathology request form is a key communication tool between clinicians and pathologists, carrying important patient and clinical details that are necessary for accurate pathological interpretation [3]. The accuracy and completeness of these forms are important for ensuring high-quality pathology services and better patient care [3].

Incomplete or missing information on histopathology request forms is a common issue in many healthcare systems [4]. Studies have shown that these problems can lead to delays in diagnosis, increased workload for pathologists, and even diagnostic errors [5]. Missing details, such as patient history, clinical findings, or laboratory results, make it harder for pathologists to provide accurate and timely reports [6]. Addressing these gaps in documentation is important for improving the efficiency and reliability of pathology services.

Clinical audits, whether internal or external, are widely used as tools to assess and improve the quality of documentation in healthcare settings. Regular audits, ideally conducted at least every six months, help ensure continuous improvement and adherence to documentation standards [7]. By evaluating current practices, identifying gaps, and implementing targeted interventions, audits can help improve compliance with documentation standards [8]. Research shows that providing training, clear guidelines, and regular feedback to clinicians can significantly enhance the quality of information recorded on histopathology request forms [8].

A complete histopathology request form should include key information, such as patient identifiers, clinical history, examination findings, laboratory results, and a provisional diagnosis [9]. This information helps the pathologist understand the clinical context and interpret the tissue findings more accurately. For example, clinical history provides important clues about the possible diagnosis, while laboratory and imaging results help support or exclude certain conditions. Incomplete forms may result in unnecessary tests or consultations, leading to delays and increased costs for both the patient and the healthcare system [8].

Efforts to improve documentation practices have included the introduction of electronic request systems, which ensure that mandatory fields are completed before submission, and the use of standardized request forms with clear instructions [10]. These measures have been shown to improve compliance and reduce errors in documentation [10]. Globally, the need for better documentation practices is recognized as a key component of quality assurance in pathology [11].

The present study was conducted with the following two primary objectives: first, to evaluate the existing documentation practices in histopathology request forms, and second, to assess the impact of targeted interventions on improving these practices. Accurate and comprehensive documentation serves as a cornerstone for ensuring reliable diagnostic outcomes, particularly in lower-middle-income countries where resource limitations and systemic challenges can impede adherence to standardized protocols. This study was undertaken in a tertiary care hospital with the aim of identifying prevalent deficiencies in documentation through an initial audit. Following this, a series of interventions were implemented, including educational sessions, dissemination of audit findings, development of standardized request forms, and ensuring their availability across all relevant departments. By comparing the documentation practices before and after the intervention, the study seeks to provide an evidence-based evaluation of the intervention's effectiveness and to propose practical recommendations for enhancing the quality and reliability of pathology services. Improving documentation practices in resource-constrained settings represents a cost-effective strategy to strengthen diagnostic accuracy, optimize patient care, and support broader healthcare quality improvement initiatives.

## Materials and methods

This study was conducted as a quality improvement project using a clinical audit framework to assess and improve the completeness of documentation on histopathology request forms at Northwest General Hospital which is a tertiary care hospital in Pakistan that provides histopathology services to various medical and surgical specialties, including gynecology, general surgery, internal medicine, oncology and allied specialities. The project employed the Plan-Do-Study-Act (PDSA) cycle methodology, a widely recognized approach for systematic quality improvement in healthcare settings [12]. The PDSA model involves iterative cycles of planning, implementing interventions, evaluating outcomes, and refining practices to achieve sustained improvement. Two audit cycles were conducted to assess the baseline performance, implement targeted interventions, and re-evaluate the outcomes to determine the effectiveness of the intervention.

PDSA cycle 1 (baseline audit)

Plan

In the initial phase, a systematic planning process was undertaken to identify deficiencies in the documentation of histopathology request forms. The research team used a data collection tool encompassing essential parameters to evaluate documentation completeness. Notably, the audit was conducted using the parameters used in the standard histopathology form employed by Newcastle upon Tyne Hospitals NHS Foundation Trust for histopathology requests.

Do

Data were collected retrospectively from 250 histopathology request forms submitted to the pathology department over a defined three-month period. All forms included in the analysis were for specimens processed within the study hospital, and forms with missing or illegible demographic information were excluded. Data collection was conducted by two independent teams, each comprising three researchers. Both teams reviewed the same set of 250 forms independently, documenting the presence or absence of information in the predefined parameters.

The parameters assessed in this study encompassed several key aspects of histopathology request form documentation. Patient identifiers were evaluated by examining the presence of essential information such as the patient's name, medical record number, age, gender, and the referring physician's name. Clinical details were reviewed for the documentation of presenting complaints, findings from physical examinations, radiological findings, laboratory investigation results, drug or medication history, and any previous cytology findings. Additionally, specimen details were assessed by noting the name of the tissue specimen, the anatomical site of the biopsy, the procedure performed, and the time of biopsy. Finally, the presence of a provisional diagnosis was evaluated, which involved recording the clinician's suspected diagnosis based on the integration of clinical, laboratory, and imaging findings.

Study and Act

The results from the two independent teams were compared, and discrepancies were resolved through consensus facilitated by a senior author with extensive experience. This approach minimized the potential for inter-observer variability and ensured data accuracy. Data were analyzed using SPSS version 26 (Armonk, NY: IBM Corp.), calculating the frequency and percentage of completed fields for each parameter. Based on the findings of cycle 1, the research team formulated an intervention strategy to address the identified gaps.

Intervention strategy

Following the analysis of the first audit cycle, an intervention was implemented to address the identified documentation deficiencies. The intervention consisted of the following components.

Presentation of Audit Findings

The findings of the first cycle were presented during a routine hospital meeting attended by consultants from various specialties, including surgery, gynecology, oncology, and internal medicine. The presentation highlighted the documentation deficiencies and emphasized their potential impact on diagnostic accuracy, treatment planning, and patient safety. Specific examples of incomplete forms were shared (with patient identifiers removed) to illustrate the practical implications of missing information.

Targeted Communication with Consultants

In addition to the group presentation, individual letters were distributed to all departmental consultants. These letters summarized the audit findings, explained the clinical importance of complete documentation, and provided clear recommendations for improvement. The letters also underscored the shared responsibility of both clinical teams and the pathology department in ensuring accurate and complete request forms.

Development of a Standardized Histopathology Request Form

In collaboration with the head of the pathology department and hospital management, a new standardized histopathology request form was developed to address the significant limitations of the existing form. The previous form lacked mandatory fields for critical patient and clinical information, leading to incomplete submissions and potential delays in processing. Additionally, it did not provide structured guidance for clinicians, increasing variability in the way requests were completed. Simply modifying the existing form would not have ensured the necessary standardization and completeness required for optimal documentation. Therefore, a new form was designed based on the histopathology request form used by the Newcastle upon Tyne Hospitals NHS Foundation Trust in the United Kingdom, incorporating mandatory fields and clear instructions to enhance accuracy, streamline workflow, and improve overall efficiency. The standardized form is attached in the appendices.

Form Distribution and Accessibility

The standardized forms were printed and distributed to all relevant clinical departments. Hospital administration ensured that adequate stocks of the new forms were maintained at all times. Additionally, clinical staff were briefed on the rationale for the new form and provided with guidance on its completion during departmental meetings and ward rounds.

PDSA cycle 2 (re-audit)

Plan

Following a three-month period of intervention implementation, the second audit cycle was planned to assess the effectiveness of the interventions. The same parameters and methodology were utilized to ensure comparability with the baseline data.

Do

A total of 150 histopathology request forms were prospectively reviewed. As in the first cycle, data collection was conducted by two independent teams, each reviewing the same 150 forms separately. The teams documented the completeness of each parameter independently, ensuring consistency and reliability.

Study

The results from the two teams were compared, and discrepancies were again resolved by the senior author. Data were analyzed using SPSS version 26, calculating the frequency and percentage of forms with complete documentation. Improvements in documentation completeness were assessed by comparing cycle 2 results with the baseline findings.

Act

The results of the second audit cycle were presented to the hospital administration and clinical departments. Based on the observed improvements, the standardized form was adopted as the hospital's official histopathology request form. Ongoing monitoring and periodic six-monthly internal re-audits were recommended to maintain and further improve documentation practices.

Data analysis

The data from both audit cycles were entered into SPSS version 26 and analyzed using descriptive statistics. For each documentation parameter, the proportion of completed fields was calculated and compared across the two cycles. Results were presented in tabular form to illustrate improvements and identify areas requiring further attention. Additionally, bar charts were used to visualize changes in documentation completeness. The study was conducted as part of an institutional quality improvement initiative. All data were anonymized, and no personally identifiable information was recorded. The intervention was educational in nature and posed no risk to patient care.

## Results

First cycle

The audit was conducted in two cycles to assess and improve the completeness of histopathology request form documentation. In the first cycle, 250 forms were retrospectively reviewed. Of these, 57% (n=143) were from female patients and 43% (n=107) were from male patients. The departmental distribution indicated that 32% (n=80) of the forms originated from the gynecology department, 47% (n=118) from the surgery and allied department, and the remaining 21% (n=52) from the medicine and allied department. While patient identifiers such as name, medical record number, age, gender, and the referring physician's name were documented in all forms, key clinical details were inconsistently recorded. A complete clinical history, defined as a comprehensive record of past medical and surgical history covering all relevant aspects, was absent from all forms, with presenting complaints documented in only 3.2% (n=8). Physical examination findings were present in 73.6% (n=184) of the forms, while radiological findings were reported in 44.4% (n=111). Laboratory investigation results were documented in 41.2% (n=103), and drug or medication history was mentioned in only 6% (n=15). Previous cytology findings were recorded in 4% (n=10) of the forms, and the last menstrual period (LMP) was documented in just 1.6% of the relevant cases (n=2).

The documentation of specimen details was similarly deficient. The name of the tissue specimen was recorded in 47.6% (n=119) of the forms, while the anatomical site was documented in 47.2% (n=118). The procedure performed was mentioned in 76.4% (n=191) of cases; however, the time of biopsy was noted in only 3.2% (n=8). A provisional diagnosis was present in 49.2% (n=123) of the forms.

Second cycle

In the second cycle, 150 histopathology request forms were reviewed after three months of the intervention. Patient identifiers remained consistently documented in 100% of the forms. Significant improvements were observed across most parameters. The documentation of a complete clinical history improved from 0% to 62% (n=93), while presenting complaints were recorded in 73% (n=110) of the forms compared to 3.2% in the first cycle. Physical examination findings increased from 73.6% to 96% (n=144), and radiological findings improved from 44.4% to 95% (n=143). Laboratory investigation results were present in 81% (n=122) of the forms compared to 41.2% previously. The documentation of drug or medication history increased from 6% to 48% (n=72), and previous cytology findings were recorded in 71% (n=107) of the forms, compared to 4% in the initial audit. For female patients, the recording of the LMP improved from 1.6% to 68% (n=102).

**Figure 1:**
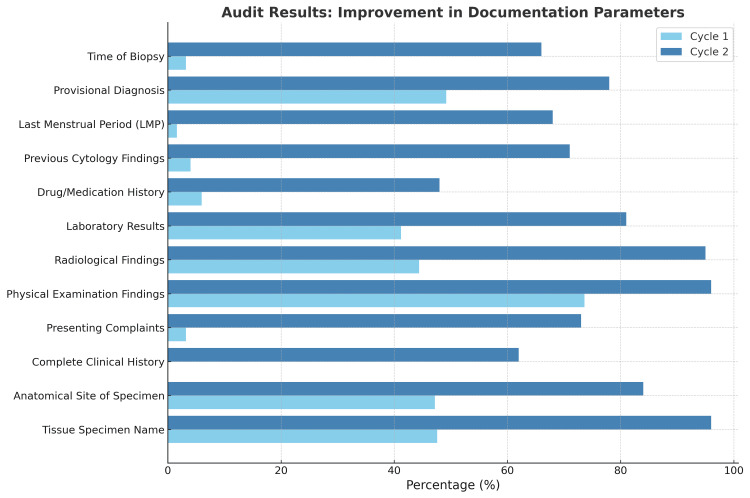
Comparison of documentation completeness across two audit cycles.

Improvements were also observed in specimen-related information. The name of the tissue specimen was documented in 96% (n=144) of the forms, an increase from 47.6%, while the anatomical site was recorded in 84% (n=126), compared to 47.2% previously. The time of biopsy, which had been noted in only 3.2% (n=8) of forms during the first cycle, was documented in 66% (n=99) in the second cycle. Additionally, the presence of a provisional diagnosis improved from 49.2% to 78% (n=117). Figure 1 demonstrates improvements in cycle 2.

Comparative analysis between the two cycles demonstrated significant improvements in all documentation parameters. Table 1 shows the comparison of documentation parameters across both audit cycles. The documentation of a complete clinical history improved by 62%, while presenting complaints increased by 69.8%. The reporting of physical examination findings improved by 22.4%, radiological findings by 50.6%, and laboratory investigation results by 39.8%. Drug or medication history documentation showed a notable increase of 42%, while previous cytology findings improved by 67%. The recording of the LMP in female patients increased by 66.4%, and the time of biopsy increased by 62.8%. Documentation of the tissue specimen name improved by 48.4%, the anatomical site by 36.8%, and the provisional diagnosis by 28.8%.

**Table 1:** Comparison of documentation parameters across both audit cycles.

Parameter	Cycle 1 (%)	Cycle 2 (%)	Improvement (%)
Patient identifiers	100	100	0
Tissue specimen name	47.6	96	48.4
Anatomical site of specimen	47.2	84	36.8
Complete clinical history	0	62	62
Presenting complaints	3.2	73	69.8
Physical examination findings	73.6	96	22.4
Radiological findings	44.4	95	50.6
Laboratory results	41.2	81	39.8
Drug/medication history	6	48	42
Previous cytology findings	4	71	67
Last menstrual period (LMP)	1.6	68	66.4
Provisional diagnosis	49.2	78	28.8
Time of biopsy	3.2	66	62.8

## Discussion

Request forms for these services play a significant role as they serve as the initial point of communication between clinicians and pathologists by providing patients' clinical data [12]. The quantity and quality of information provided in the request form determine the proper testing and the accuracy of the findings. Inadequate filling of the request forms increases the risk of diagnostic errors and wastes time due to the requirement for further communication between the doctor and histopathologist [13]. Literature shows that diagnostic errors can lead to financial burden, resource dissipation, and increased morbidity and mortality [14]. Previous studies on surgical record-keeping, such as those utilizing the STAR and CRABEL scoring systems, have emphasized that structured documentation reduces errors and enhances compliance with standardized medical record practices [15,16]. While these tools are primarily used in surgical audits, their core principles of structured data collection and audit cycles can be applied to histopathology request forms to improve documentation practices.

Patient identification is crucial to the correct labeling of diagnosis, on which decisions regarding further workup and management of the patient are made. Failure to correctly document the patient identifiers, which include patient’s name, age, gender, and medical record (MR) number, can result in serious harm and care delay [17]. In both audit cycles of our quality improvement project, all patient identifiers were correctly documented in all cases showing excellent compliance with the medical practice. In a study done in Nigeria by Nwoga et al., patient’s name was documented in all cases, while age, gender, and MR number were documented in 52%, 97%, and 94% of the cases, respectively [18]. In another study done by Siddiqui et al., reported results showed that the patient’s name was documented in all cases, while the MR number was mentioned only in 64% of the cases, which can lead to mislabeling of diagnosis [19]. Similar challenges in record-keeping have been identified in audits using the CRABEL score, where missing patient identifiers were a major factor affecting documentation completeness in surgical case records [16].

Histopathology specimens establish the tissue diagnosis of the patient and are crucial in determining the treatment and follow-up strategies [20]. Mentioning the name of the tissue specimen is important as it helps the pathologist to correctly diagnose the condition. In the first cycle, name of tissue specimen was mentioned in 47.6% of the cases, and it significantly increased to 96% in the second cycle after intervention. This jump of 48.4% between two cycles shows the importance of standardized intervention and its robust implementation. Anatomical site from where the tissue specimen has been collected is also of prime importance, as different diseases carry variable significance as the anatomical position changes [21]. Defective or missed identification of the anatomical location of tissue is a major category of error faced in interpretation of tissue specimens [21]. A notable improvement of 36.8% was seen in documentation of anatomical sites between two audit cycles showing compliance with the intervention. This aligns with findings from STAR-based surgical audits, where detailed operative records, including site identification, showed significant improvements following standardized documentation practices [15].

A structured and systematic approach to history-taking and its documentation increases a clinician's ability to make informed clinical judgments and critical decisions, thereby ensuring patient safety and effective treatment [22]. Documentation of clinical history and presenting complaints of the patient on the histopathology request forms aids in the correct diagnosis on which future treatment plan depends [22]. Our study found a complete lack of documented clinical history in cycle 1, which likely reflects systemic issues such as the absence of standardized documentation protocols, time constraints on clinicians, and a reliance on verbal communication rather than written records. Compared to studies like Shinde and Dhanve, where only 3.8% of cases had inadequate clinical history documentation, our findings suggest that filling out this section was not a routine practice among referring clinicians [23]. Institutional factors, such as differences in workload pressures, clinician awareness, and varying documentation practices, may have contributed to these discrepancies. The low documentation rate for laboratory results (41.2%) further supports the idea that essential clinical details were not consistently included in histopathology request forms. These gaps highlight the need for structured interventions, such as standardized forms and targeted training, to reinforce the importance of comprehensive documentation and improve compliance across departments. STAR and CRABEL-based audits in surgical settings have also identified incomplete clinical history as a barrier to effective patient management, reinforcing the need for structured documentation interventions [15,16].

Physical examination of the patient and its documentation holds a cornerstone in identifying the correct diagnosis of the patient [23]. It provides a wider view to the pathologist who can use this important data of the patient and label the specimen with more accuracy. In the first cycle, 73.6% of the physical examination findings were noted down in the histopathology request forms, while in the second cycle, this number increased to the documentation in 96% of the cases. This shows the commitment of clinicians to adhere to the performance of physical examination and documentation of its findings in histopathology request forms.

Histopathologists do not see patients in person and rather rely on the patient data provided in the request form [24]. Radiology findings are known to enhance the value of provided clinical data and assist in determining the correct diagnosis [24]. During the first and second audit cycles, a major increase of 50.6% was seen in the documentation of radiology findings, while, in contrast, a study done by Abbasi et al. showed adherence to this parameter in only 5.2% of all the cases [1,25]. Laboratory test results also provide useful insight into the suspected diagnosis of the condition. In the same audit, laboratory findings were conveyed to the histopathologist in 12.4% of the cases, while in our two cycles of audits, these numbers stood at 41.2% and 81%, showing the robustness of compliance and resulting in marked improvement [26]. Similarly, documentation of drug/medication history, last menstrual period (LMP), and previous cytology findings improved by 42%, 66%, and 67%, respectively. Despite this betterment, the implementation of digital electronic system can provide an error-free service of filling these details automatically.

The clinician and the medical team, being the first to contact the patient, have the complete picture in front of them, from a detailed history to physical exams and laboratory findings. They can provide a preliminary diagnosis on which the histopathologist can work [26]. In our two audit cycles, documentation of provisional diagnosis and time of biopsy witnessed a 28.8% and 62.8% improvement, respectively. Time of biopsy is also an important data variable that a histopathologist can correlate with the patient’s history and timeline of symptoms, furthering the accuracy of the final diagnosis. Structured auditing systems such as STAR and CRABEL have demonstrated similar improvements in surgical record-keeping, reinforcing the importance of standardized documentation in clinical practice [15,16].

Histopathology request forms serve as a critical communication bridge between clinicians and histopathologists, and deficiencies in their documentation can impact diagnosis, treatment planning, and patient follow-up [26]. While our study focused on improving the completeness of histopathology request forms, the principles of structured auditing seen in STAR and CRABEL scoring systems could be applied to further enhance documentation quality in this area. Just as these tools have successfully standardized and improved surgical record-keeping through systematic audits and structured interventions, a similar framework could be developed for histopathology request forms. Implementing a scoring-based auditing system tailored for histopathology request forms would allow for quantifiable assessment of documentation quality, helping institutions identify areas for improvement and track progress over time. Future initiatives could incorporate digital electronic systems alongside structured audits inspired by STAR and CRABEL to create a more standardized, efficient, and error-free approach to histopathology documentation. The frequency of internal audits plays a crucial role in maintaining and sustaining these improvements, as regular monitoring ensures continued adherence to documentation standards and helps identify areas requiring further intervention. Encouraging interdisciplinary collaboration between clinicians and pathologists, periodic audits, and training programs will further reinforce best practices, ultimately reducing diagnostic errors and improving patient safety.

One of the key strengths of our study is its systematic audit approach, which effectively identified gaps in histopathology request form documentation and demonstrated significant improvements following intervention. The use of quantitative metrics allowed for objective assessment of compliance, making the findings replicable and applicable to other institutions aiming to enhance documentation quality. Additionally, our study highlights the importance of structured interventions, reinforcing the value of standardization in medical documentation. However, a limitation of our study is that it focused solely on histopathology request forms without incorporating a formalized scoring system like STAR or CRABEL, which have been successfully used to evaluate documentation in surgical and case records. While our study provides valuable insights into improving record-keeping practices, future research could explore the development of a scoring framework tailored to histopathology request forms, similar to STAR and CRABEL, to allow for more structured evaluations and longitudinal tracking of improvements. Furthermore, this study was conducted in a single institution, which may limit its generalizability; however, the findings still provide a strong foundation for broader implementation of structured auditing and digital interventions to enhance documentation quality.

## Conclusions

This study demonstrated that using the PDSA cycle significantly improved the completeness of histopathology request form documentation at a tertiary care hospital. The introduction of a standardized form, clinician engagement, and regular feedback led to notable improvements in key documentation areas, supporting better communication between clinicians and pathologists. These findings highlight the importance of structured quality improvement initiatives in enhancing diagnostic accuracy and patient care. To maintain these improvements, ongoing audits, clinician training, and the adoption of digital documentation systems should be considered. While this study was conducted at a single center with a limited sample size in the second cycle, its approach can be applied in similar healthcare settings to strengthen documentation practices and improve patient outcomes.

## Figures and Tables

**Figure 2:**
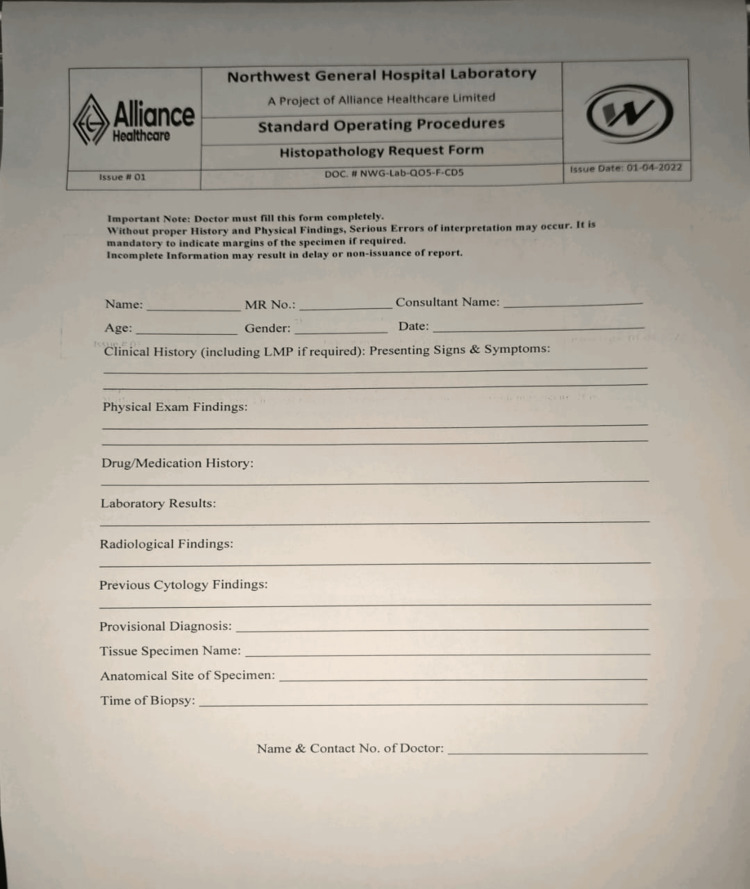
Standardized histopathology request form.

## References

[1] Abbasi F, Asghari Y, Niazkhani Z (2023). Information adequacy in histopathology request forms: a milestone in making a communication bridge between confusion and clarity in medical diagnosis. Turk Patoloji Derg.

[2] Morelli P, Porazzi E, Ruspini M, Restelli U, Banfi G (2013). Analysis of errors in histology by root cause analysis: a pilot study. J Prev Med Hyg.

[3] Romano RC, Novotny PJ, Sloan JA, Comfere NI (2016). Measures of completeness and accuracy of clinical information in skin biopsy requisition forms: an analysis of 249 cases. Am J Clin Pathol.

[4] Esposito P, Canton AD (2014). Clinical audit, a valuable tool to improve quality of care: general methodology and applications in nephrology. World J Nephrol.

[5] Jedwab RM, Franco M, Owen D, Ingram A, Redley B, Dobroff N (2022). Improving the quality of electronic medical record documentation: development of a compliance and quality program. Appl Clin Inform.

[6] Elhassan EA, Khalifa MS, Ibrahim F, Mohammed SA (2024). Adequacy of histopathology request forms and specimens sent to two histopathology centers in Khartoum, Sudan. Surg Exp Pathol.

[7] Koh J, Ahmed M (2021). Improving clinical documentation: introduction of electronic health records in paediatrics. BMJ Open Qual.

[8] Nkengasong JN, Birx D (2014). Quality matters in strengthening global laboratory medicine. Afr J Lab Med.

[9] Taylor MJ, McNicholas C, Nicolay C, Darzi A, Bell D, Reed JE (2014). Systematic review of the application of the plan-do-study-act method to improve quality in healthcare. BMJ Qual Saf.

[10] Patel K, Chotai N (2011). Documentation and records: harmonized GMP requirements. J Young Pharm.

[11] Sloan P, Robinson M (2020). Quality assessment across disciplines in head and neck cancer treatment diagnostic pathology in HNSCC. Front Oncol.

[12] Bonini P, Plebani M, Ceriotti F, Rubboli F (2002). Errors in laboratory medicine. Clin Chem.

[13] Oyedeji OA, Ogbenna AA, Iwuala SO (2015). An audit of request forms submitted in a multidisciplinary diagnostic center in Lagos. Pan Afr Med J.

[14] Carraro P, Plebani M (2007). Errors in a stat laboratory: types and frequencies 10 years later. Clin Chem.

[15] Kakada P, Ramalingam K, Ramani P, Krishnan M (2024). Assessment of the quality of oral squamous cell carcinoma clinical records in oral surgery with Surgical Tool for Auditing Records (STAR) scoring. BMC Oral Health.

[16] Aarthi S, Ramalingam K, Ramani P, Krishnan M (2024). CRABEL score assessment for oral surgery excision biopsy case notes of oral squamous cell carcinoma. Cureus.

[17] Lippi G, Mattiuzzi C, Bovo C, Favaloro EJ (2017). Managing the patient identification crisis in healthcare and laboratory medicine. Clin Biochem.

[18] Nwoga MC, Okwuosa CU, Uzosike CC (2018). Oral and maxillofacial biopsies: a 6-year audit of histopathology forms. Afr J Oral Maxillofac Pathol Med.

[19] Siddiqui MS, Kumar S, Kumar D, Khalid S, Hassan W, Khatri M (2024). Are we providing enough information to the pathologists? An audit of filling of histopathology request forms for surgically resected tumours. J Coll Physicians Surg Pak.

[20] (2004). Histopathology Specimen: Clinical, Pathological and Laboratory Aspects. London: Springer.

[21] Karki S (2015). Errors: detection and minimization in histopathology laboratories. J Pathol Nepal.

[22] Peart P (2022). Clinical history taking. Clin Integr Care.

[23] Shinde SV, Dhanve MJ (2021). Audit in surgical histopathology at a tertiary healthcare center: study of preanalytical and analytical phase. Indian J Pathol Microbiol.

[24] Clark BW, Niessen T, Apfel A, Luckin J, Lee YZ, Desai SV, Garibaldi BT (2022). Relationship of physical examination technique to associated clinical skills: results from a direct observation assessment. Am J Med.

[25] Ali SM, Kathia UM, Gondal MU, Zil-E-Ali A, Khan H, Riaz S (2018). Impact of clinical information on the turnaround time in surgical histopathology: a retrospective study. Cureus.

[26] Keshwar S, Jain N, Raut T, Singh V, Shrestha A (2024). A retrospective analysis of concordance between clinical and histopathologic diagnoses and completeness of oral biopsy forms at a tertiary dental hospital in Eastern Nepal. Int J Dent.

